# Predictive value of modified frailty index-5 to major complications after videothoracoscopic pulmonary resections

**DOI:** 10.1007/s13304-025-02232-y

**Published:** 2025-05-14

**Authors:** Muhammet Sayan, Mahir Fattahov, Fevzi Oguzhan Temirkaynak, Nazmiye Koska, Bengisu Artiran, Muhammet Tarik Aslan, Gunel Ahmadova, Aysegul Kurtoglu, Irmak Akarsu, Ismail Cuneyt Kurul, Ali Celik

**Affiliations:** Department of Thoracic Surgery, Gazi University, 06560 Ankara, Turkey

**Keywords:** Modified frailty index 5, VATS, Lobectomy, Complication, Postoperative

## Abstract

Although minimally invasive methods have become widespread, pulmonary resections due to lung cancer continue to be an important cause of postoperative morbidity. Herein, we have investigated the predicting efficacy of modified frailty index-5 (MFI-5) for postoperative complications in patients who underwent pulmonary resection by VATS for non-small cell lung cancer (NSCLC). We retrospectively reviewed the data of patients who underwent VATS lobectomy/segmentectomy for NSCLC. MFI-5 score was calculated according to hypertension, diabetes mellitus, chronic obstructive pulmonary disease, congestive heart failure, and functional independence status. Major postoperative complications were determined based on Clavien–Dindo classification. The predictive efficacy of MFI-5 score for major complications was tested by univariate and multivariate logistic regression analysis. A total of 336 patients were included in the study. The mean age was 65.6 ± 9.8 years. MFI-5 score was zero in 126 (37.5%) patients and positive in 210 patients. The major complication rate was 25.9%. Multivariate analysis showed that 2 and higher MFI-5 score significantly predicted the presence of postoperative major complications (*p*: 0.004, OR: 4.3, 1.58–12.5 95% CI). The MFI-5 score can significantly predict the presence of major postoperative complications, including 30-day mortality, in patients undergoing VATS pulmonary resection for NSCLC. *Clinical registration* 2024–324, approved by Gazi University Local Ethics Committee.

**Graphical abstract:**

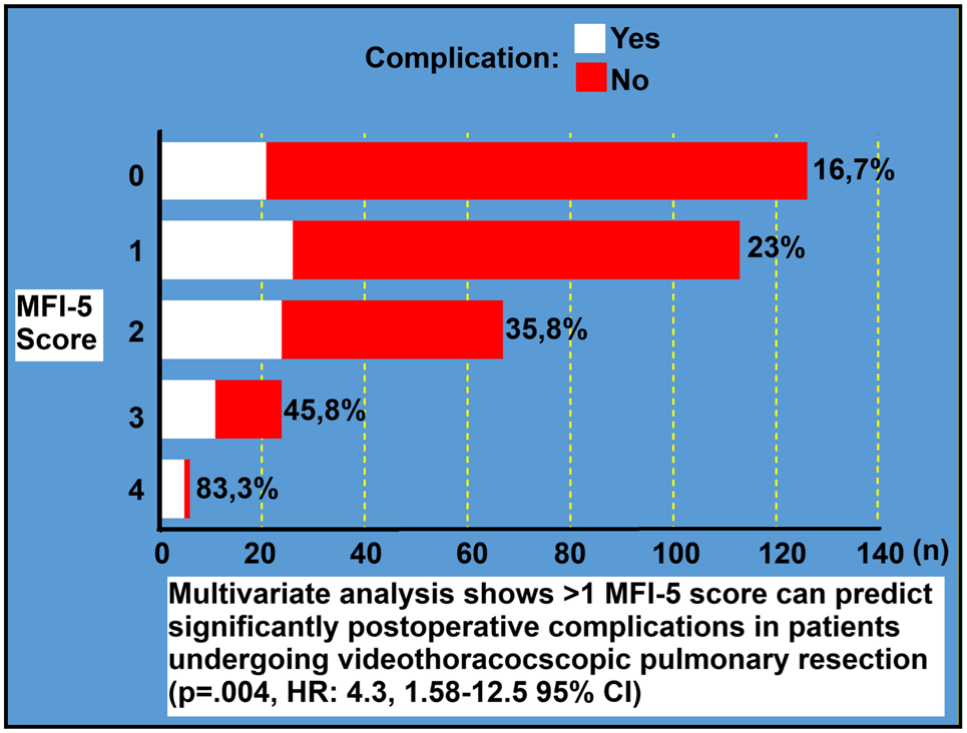

## Introduction

Lung resections by video-assisted thoracic surgery (VATS) are currently being successfully performed in early-stage non-small cell lung cancer (NSCLC). Although less common than thoracotomy, serious postoperative complications can also occur in VATS pulmonary resections. These complications can result in non-cancer-related deaths. Various indices predicting postoperative complications have been of interest to clinicians for years. Frailty index is one of them and frailty has been defined as a biological syndrome in which the resistance and reserve to stressors decrease with age. This syndrome results from cumulative declines in multiple physiological systems [1]. When the frailty index was first proposed, it included more than 70 parameters. Difficulties in data collection and analysis led clinicians to simplify the index, and it was first reduced to 11, then to 5 parameters, and was named modified frailty index-5 (MFI-5). The effectiveness of MFI-5 was demonstrated with comparative studies. The items of MFI-5 are hypertension (HT), diabetes mellitus (DM), chronic obstructive pulmonary disease (COPD), congestive heart failure (CHF), and functional independence status (FS) [2]. The presence of HT and antihypertensive drugs used in treatment may lead to postoperative complications. It has been reported that patients using angiotensin-converting enzyme inhibitors or angiotensin receptor blockers have an increased risk of fatal postoperative complications such as acute renal failure, acute coronary syndrome, or stroke when they undergo surgery. The probable cause of these adverse events is sudden intraoperative hypotension and perfusion problems after anesthesia induction [3]. The presence of HT requiring antihypertensive medication was reported as positive in MFI-5 [4]. DM is a well-known entity that negatively affects wound healing and has immunosuppressive effects. In pathophysiology, uncontrolled hyperglycemia causes microvascular damage by macrophages and neutrophils and long-term systemic changes affect pulmonary function. In addition, hyperglycemia causes prolonged hypercatabolism and impaired collagen tissue formation. In addition, hyperglycemic patients are more susceptible to respiratory tract infections caused by atypical microorganisms and are at increased risk of postresection pneumonia, bronchopleural fistula, pyothorax, and mucus plugs in the airways. In addition, surgical stress, catabolism, and corticosteroids that may be required postoperatively may further worsen glycemic control [5]. COPD is an important risk factor for postoperative complications in other surgeries, while the risk becomes more pronounced in pulmonary resections. Since smoking is a common risk factor for both lung cancer and COPD, the presence of COPD in patients who are candidates for lung resection is not unusual. Impaired pulmonary parenchymal structure can lead to serious complications, especially postoperative prolonged air leak, bronchopleural fistula, respiratory failure, and postoperative pneumonia. The studies have reported that the presence of emphysema is an independent poor prognostic factor for overall survival in patients undergoing lung resection [6]. CHF is a significant cause of morbidity, hospital readmission, and mortality after pulmonary resections [7, 8]. FS is the ability of individuals to perform daily activities such as self-care, toileting, walking, etc*.* without the need for assistance. Dependent FS is essentially an indirect indicator of conditions such as dementia, stroke sequelae, advanced age, and severe respiratory failure. It has been reported that patients with dependent FS significantly tend to have postoperative complications [9].

Although the predictive significance of MFI-5 in various surgeries has been demonstrated, studies involving pulmonary resections are quite scarce. In this study, we investigated the effect of MFI-5 in predicting postoperative complications, including 30-day mortality, in patients who underwent VATS lobectomy or segmentectomy for NSCLC.

## Materials and methods

### Patient selection

**Fig. 1:**
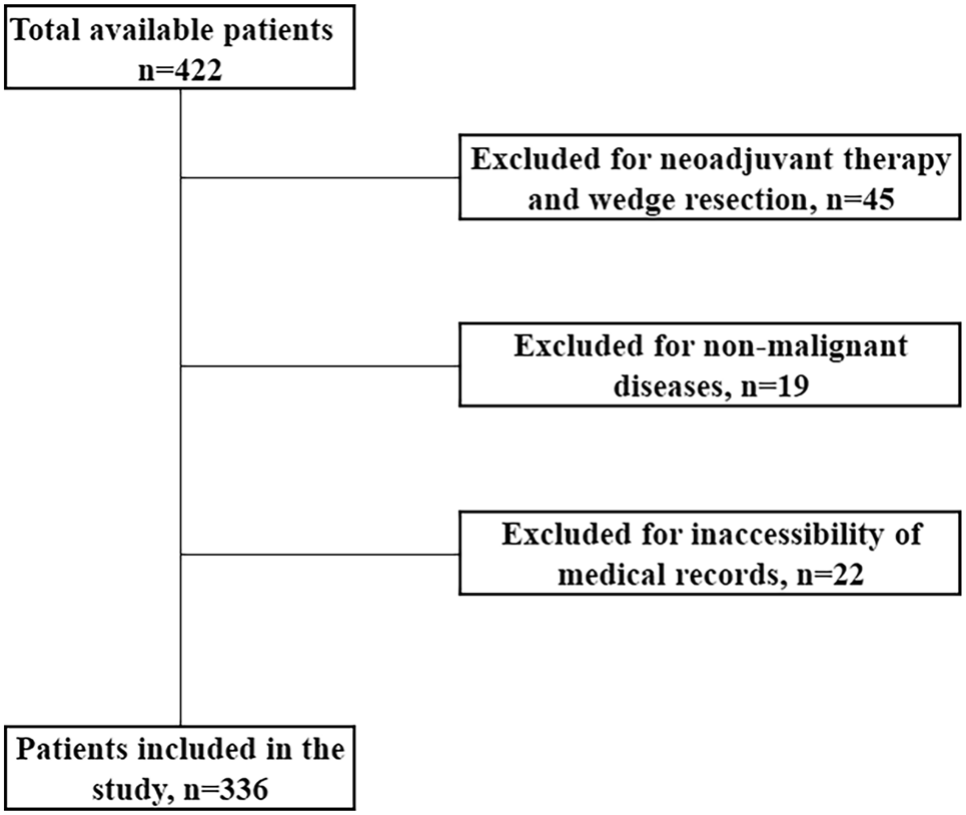
A CONSORT diagram showing patient selection

Following the approval of the Gazi University Ethics Committee with 2024-324 registration number, the records of patients who underwent VATS-pulmonary resection for NSCLC between January 2018 and December 2023 were retrospectively reviewed. Written informed consent was obtained from all patients and/or their legal guardians for the use of their data in a scientific study. The study procedure was conducted in accordance with the ethical standards of the Declaration of Helsinki. Patients who underwent lobectomy or anatomic segmentectomy without neoadjuvant therapy were included in the study. Patients who underwent wedge resection, those who underwent pulmonary resection for metastatic or non-malignant diseases, those who did not undergo lymph node dissection, and those whose records could not be accessed were excluded from the study. The wedge resection procedure was not included in the study because it is a relatively less complicated procedure compared to anatomic resections. Figure 1 shows a CONSORT diagram of patient selection. Data on age, gender, tumor diameter, tumor histopathology, MFI-5 items, the operation performed, tumor location, major complications, presence of postoperative complications, and death within the first 30 days of postoperative surgery were collected. The presence of major complications was defined as grade 2 and above according to the Clavien–Dindo classification [10].

### Covariates

We defined major complications in patients as pneumonia requiring noninvasive/invasive mechanical ventilation or ECMO support, empyema, atrial fibrillation, broncho-pleural fistula, bleeding requiring blood transfusion or revision, acute renal failure, surgical readmission to intensive care or ward, chylothorax, prolonged air leak and death within 90 days postoperatively. We classified surgical procedures as lobectomy and anatomic segmentectomy. MFI-5 items were defined as DM, HT, COPD, CHF, and FS based on previous studies [11]. We marked the HT score as positive for patients requiring antihypertensive medication. To determine the DM score, we based the HbA1 C value, the patient’s medical history, and the endocrinologist’s opinion. For a positive COPD score, we considered the patient's medical history, medications, pulmonary function tests, or medical reports from the Chest Disease Consultant. We recorded the CHF score according to the presence of heart failure, confirmed by echocardiography findings or a cardiology specialist examination. We defined the FS score as positive in patients who cannot independently perform daily activities such as eating, walking, bathing, etc., due to some pathologies such as stroke sequelae, dementia disorder, and cardiopulmonary insufficiency.

### Statistical analysis

All statistical analyses were performed using SPSS 25.0 (IBM Corp., Armonk, NY, USA) software. The categorical variables were given as n and %. The distribution normality of numerical variables was determined by the Kolmogorov–Smirnov test and given as mean and standard deviation or median and range according to distribution normality. The predictive power of specific variables (age, gender, operation side, tumor stage, tumor diameter, MFI-5 score) for postoperative major complications was investigated by univariate and multivariate binary logistic regression analysis. To avoid the effect of multicollinearity, MFI-5 score was calculated separately for 1 and 2 as a covariate for multivariate analyses. Any correlation between MFI-5 scores and age was investigated by Pearson Chi-Square or Fisher’s Exact Test according to the expected count. Analyses were performed with a 95% confidence interval. A *p* value of less than 0.05 was considered statistically significant.

## Results

**Fig. 2:**
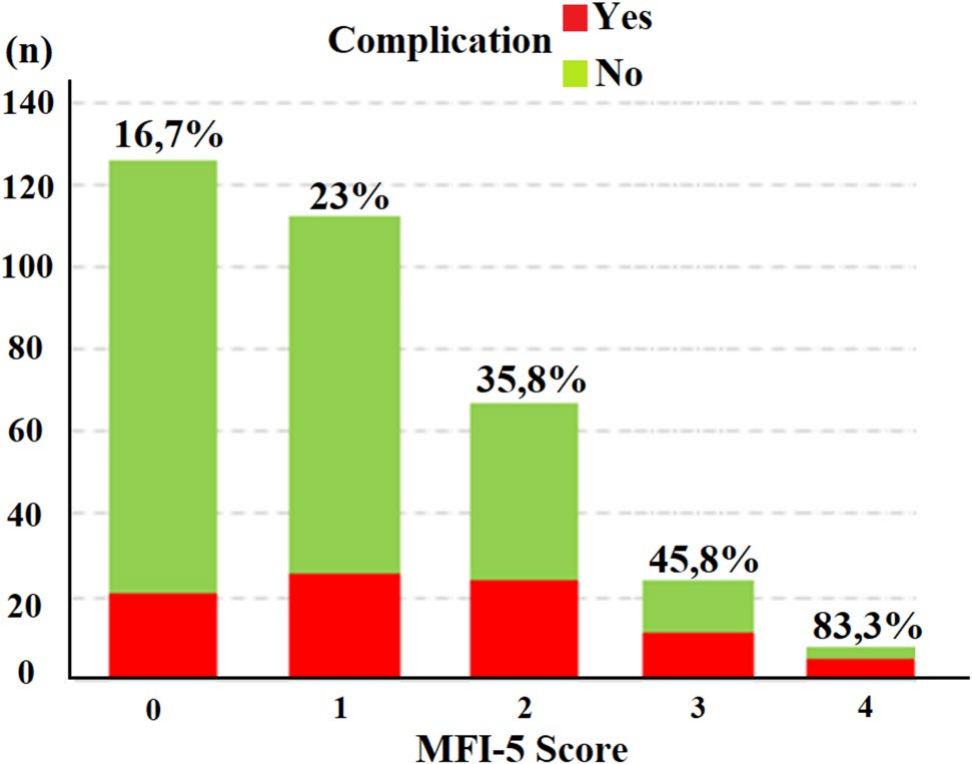
The figure shows the complication rates according to the MFI-5 score. *MFI*−5 modified frailty index-5

A total of 336 patients who met the study criteria were included. 109 (32.4%) of the patients were female and the mean age was 65.6, SD: 9.8. All patients included in the study underwent a bi-portal VATS pulmonary resection technique. The most performed surgery was the right upper lobectomy in 79 patients (23.5%). The most common histopathology was adenocarcinoma in 198 patients (58.8%). There were major postoperative events in 87 patients (25.9%) and the most common complication was prolonged air leak in 34 patients (10.1%). Mortality occurred in the first 30 days in four patients (1.2%). The MFI-5 score was 0 in 126 patients (37.5%) and 1 in 113 patients (33.6%), and the most common MFI-5 item was HT in 139 patients (41.4%). The clinicopathological characteristics of the patients are given in Table 1. According to univariate analyses, MFI-5 scores ≥ 1 and ≥ 2 (*p* = 0.003 and < 0.0001 respectively), HT (*p* = 0.01), CHF (*p* = 0.03), and COPD (*p* = 0.02) were found to be significant predictors for postoperative complications, while multivariate analyses showed that only ≥ 2 MFI-5 score was significant (*p* = 0.004, OR: 4.3, 1.58–12.5 95% CI). The p value was 0.8 by Hosmer–Lemeshow of our model in multivariate analysis. Table 2 shows the univariate and multivariate analysis results according to various variables. The patients were stratified according to age 65 and 70 years and we detected older age correlated with positive MFI-5 score by Pearson chi-square or Fisher’s exact tests (*p* < 0,001, Table 3). Although the univariate analysis showed a significant association between age and complications, the multivariate analysis did not. Comparative analyses showed that complications were detected in 16.7% of patients with an MFI-5 score of 0, while the complication rate in patients with scores of 1, 2, 3, and 4 was found to be 23%, 35.8%, 45.8%, and 83.3%, respectively (Fig. 2; Table 4).

**Table 1:** Characteristics of patients included in the study, n = 336

				n	%
Age (Mean ± SD)		65.6 ± 9.8			
Tumor Diameter (Median with min–max)		2.1 (0.1–7)			
LOS-ICU (Median with min–max)		1 (1–8)			
LOS-Hospital (Median with min–max)		7 (2–30)			
Gender	Female			109	32.4
	Male			227	67.6
Side	Right			178	53
	Left			158	47
Surgery	Lobectomy			289	86.0
		RUL		79	23.4
		RUSL		3	0.9
		RML		25	7.4
		RLL		46	13.7
		RLSL		2	0.6
		Bilobectomy		6	1.8
		LUL		72	21.4
		LUSL		1	0.3
		LLL		55	16.4
	Segmentectomy			47	14.0
		RS1		4	1.2
		RS2		5	1.5
		RS3		1	0.3
		RS6		2	0.6
		RS7		2	0.6
		RS1 + 3		1	0.3
		RS10		2	0.6
		LS1 + 2		12	3.6
		LS3		2	0.6
		LS4 + 5		6	1.8
		LS6		2	0.6
		LS8		3	0.9
		LS10		2	0.6
		LS9		1	0.3
		LS1 + 2 + 3		2	0.6
N Status		N0		260	77.4
		N1		31	9.2
		N2		45	13.4
VPI		Yes		81	24.1
		No		255	75.9
Histopathology		Adenocarcinoma		198	58.9
		Squamous CA		77	22.9
		Adenosquamous CA		6	1.8
		Neuroendocrine Tumors		40	11.9
		LCC		7	2.1
		Sarcomatoid CA		6	1.8
		ACC		2	0.6
MFI-5 Items					
		HT		139	41.4
		CHF		28	8.3
		DM		100	29.8
		COPD		38	11.3
		FS		6	1.8
MFI-5 Score	0			126	37.5
	1			113	33.7
	2			67	19.9
	3			24	7.1
	4			6	1.8
Complication	No			249	74.1
	Yes			87	25.9
		Pneumonia/respiratory failure	CD 2-3a-4	10	3.0
		AF	CD 2	11	3.3
		Chylothorax	CD 3a-b	4	1.2
		Empyema	CD 3a	3	0.9
		BPF	CD 3a-4	1	0.3
		Bleeding	CD 2-3a-4	9	2.7
		Revision	CD 3b-4	4	1.2
		Death	CD 5	4	1.2
		ARF	CD 3a-3b	9	2.7
		Readmission to Hospital	CD 2-3a-4	12	3.6
		Prolonged Air Leak	CD 3a-4	34	10.1
		Readmission to ICU	CD 4	15	4.5

*ACC* adenoid cystic carcinoma, *AF* atrial fibrillation, *ARF* acute renal failure, *CA* carcinoma, *CD* Clavien Dindo grade, *CHF* congestive heart failure, *COPD* chronic obstructive pulmonary disease, *DM* diabetes mellitus, *FS* functional status, *HT* hypertension, *ICU* intensive care unit, *LCC* large cell carcinoma, *LOS* length of stay, *LLL* left lower lobectomy, *LUL* left upper lobectomy, *LUSL* left upper sleeve lobectomy, *MFI* modified frailty index, *RLL* right lower lobectomy, *RLSL* right lower sleeve lobectomy, *RML* right middle lobectomy, Sleeve Lobectomy, *RUL* right upper lobectomy, *RUSL* right upper sleeve lobectomy

**Table 2:** Results of univariate and multivariate analysis according to some covariates

Covariates	Univariate			Multivariate		
	*p* value	OR	CI (95%)	*p* value	OR	CI (95%)
Age	0.01	1.04	1.02–1.07	0.1	1.1	0.98–1.05
Side	0.6	1.1	0.69–1.84	–	–	–
Gender	0.3	0.7	0.46–1.35	–	–	–
Tumor diameter	0.2	0.9	0.78–1.03	0.6	1.1	0.87–1.25
Type of surgery	0.6	1.1	0.56–2.40	–	–	–
Tumor stage	0.6	0.8	0.46–1.65	–	–	–
MFI-5 score 0 vs > 0	0.003	2.3	1.35–4.16	0.5	1.4	0.55–2.94
MFI-5 score 0 vs 1 (excluded 2–3–4)	0.2	1.5	0.78–2.85	0.1	2.2	0.81–6.42
MFI-5 score < 2 vs ≥ 2	< 0.0001	2.9	1.72–4.8	0.004	4.3	1.58–12.5
HT	0.01	1.8	1.1–3.12	0.3	1.6	0.58–4.56
DM	0.1	1.4	0.85–2.42	0.07	2.2	0.92- 5.26
CHF	0.03	2.3	1.05–5.1	0.08	3.0	0.83–5.37
COPD	0.02	2.9	1.49–5.96	0.1	2	0.74–6.77
FS	0.07	3.9	0.86–18.0	0.1	5	0.61–33.3

*CHF* congestive heart failure, *CI* confidence interval, *COPD* chronic obstructive pulmonary diseases, *DM* diabetes mellitus, *FS* functional status, *HT* hypertension, *MFI* modified frailty index, *OR* odds ratio

**Table 3:** Crosstabs of MFI-5 score according to the age by Pearson Chi-Square or Fisher’s Exact Test, *N* = 336

MFI-5 score	Age < 65 (*n*)	Age ≥ 65 (*n*)	*p* value	Age < 70 (*n*)	Age ≥ 70 (*n*)	*p* value
0 vs ≥ 1						
0	85/78	41	< 0.001	103	23	< 0.001
1–4	78	132		123	87	
0 vs 1						
0	85	41	< 0.001	103	23	< 0.001
1	37	76		67	46	
< 2 vs ≥ 2						
0–1	122	117	0.1	170	69	0.02
2–4	41	56		56	41	
< 3 vs ≥ 3						
0–2	150	156	0.5	210	96	0.09
3–4	13	17		16	14	
< 4 vs ≥ 4						
0–3	161	169	0.4	224	106	0.08
4	2	4		2	4	

*MFI*−5 modified frailty index-5

**Table 4:** Crosstabs of major complications and some covariates

	Total n	Complication: yes *n* and (%)	Complication: no *n* and (%)
MFI-5 score			
0	126	21 (16.7%)	105 (83.3%)
1	113	26 (23%)	87 (77%)
2	67	24 (35.8%)	43 (64.2)
3	24	11 (45.8%)	13 (54.2%)
4	6	5 (83.3%)	1 (16.7%)
5	–		
Age			
< 65 years	163	34 (20.9%)	129 (79.1%)
≥ 65 years	173	53 (30.6%)	120 (69.1)
Hypertension			
Yes	139	46 (33.1%)	93 (66.9%)
No	197	41 (20.8%)	156 (79.2%)
DM			
Yes	100	31 (31%)	69 (69%)
No	236	56 (23.7%)	180 (76.3%)
COPD			
Yes	38	18 (47.4%)	20 (52.6%)
No	298	69 (23.2%)	229 (76.8%)
CHF			
Yes	28	12 (42.9%)	16 (57.1%)
No	308	75 (24.4%)	233 (75.6%)
Functional status			
Dependent	7	4 (57.1%)	3 (42.9%)
Independent	329	83 (25.2%)	246 (74.8%)
Type of surgery			
Segmentectomy	47	11 (23.4%)	36 (76.6%)
Lobectomy	289	76 (26.3%)	213 (73.7%)
N Status			
N0	260	65 (25.0%)	195 (75%)
N1	31	11 (35.5%)	20 (64.5%)
N2	45	11 (24.4%)	34 (75.6%)

*CHF* congestive heart failure, *COPD* chronic obstructive pulmonary disease, *DM* diabetes mellitus, *MFI*−5 modified frailty index 5, *N* lymph node metastasis

## Discussion

The present study demonstrated the effectiveness of MFI-5 in predicting postoperative complications in patients undergoing VATS-pulmonary resection for lung cancer. While there are studies in the literature investigating the relationship between MFI-5 and the occurrence of postoperative complications in various types of cancer, those related to lung cancer are quite few [2]. Khalid et al. showed that the risk of postoperative complications was significantly increased, and survival was significantly worse in patients with MFI-5 scores greater than two who underwent pancreatoduodenectomy due to pancreatic ductal adenocarcinoma [12]. Similarly, in our study, the risk of postoperative complications was significantly higher in patients with MFI-5 scores greater than 1, but since it included different cancer stages, a comparison between MFI-5 scores and survival could not be made. In the study conducted by Kelley et al., the risk of postoperative complications in patients with ovarian cancer who underwent hyperthermic intraperitoneal chemotherapy was found to be high in patients with MFI-5 scores greater than 2, and the MFI-5 score was also found to be correlated with age [13]. In our study, we found a similar correlation between age and an MFI-5, and we concluded that the MFI-5 score is a significant predictor of postoperative events. However, multivariate analysis showed that age alone is not a significant prognostic factor, indicating that the presence and number of comorbidities are the real determinants of complications. Demirci et al. demonstrated that a high MFI-5 score is associated with increased postoperative complications and suggested that the score is an effective factor in treatment selection [14]. Since our study was retrospective and patients who received neoadjuvant treatment were not included, an analysis regarding treatment selection was not performed. It is expected that frailty and related scores will rise with age and that the increased risk of postoperative complications at older ages may be due to comorbidities [1]. The results of the univariate analysis of our study between postoperative complications and age, as well as crosstabs between age (65 and 70 years) and MFI-5 score, suggest that age is an important factor for postoperative events. However, age was no statistically significant factor in our multivariate analyses. This dilemma may be due to age stratification in the Pearson Chi-square test or clustering of data in univariate binary logistic regression analyses, or both.

Studies on the relationship between complications after lung resections and MFI-5 score are scarce in the literature. Tamburini et al. showed that patients with a high fragility index had significantly higher hospital readmission and postoperative complication rates after lung resection, and overall survival was significantly worse in these patients in the propensity score analysis [15]. In our study, the hospital readmission rate was 3.5% and the intensive care unit readmission was 4.5%. A study investigating the effect of the modified frailty index in patients undergoing pulmonary resection for NSCLC showed three times increased risk of complications in those with an MFI score of 2 or more [11]. In a large database study, including 36,587 patients, by Lee et al. demonstrated that MFI-5 was a significant predictor for postoperative complications, and its predictive power was similar to the American Society of Anesthesiologists and the Charlson Comorbidity Index. In the study, extended resections such as extra-pleural pneumonectomy and carinal resection were excluded, and the surgical method (minimally invasive, robotic, or thoracotomy) was not analyzed [4]. Although our study is similar regarding results, it differs in that only VATS resections were included.

Our study had some limitations. First, it was a retrospective study and included a relatively small number of patients. Another limitation is that due to the number of cases, we could not analyze the risk of developing complications separately according to the degree of MFI-5 items; we could only score the factor as present or absent. Another limitation is that due to the low number of cases, a separate analysis of the MFI-5 score’s predictive power for 30-day postoperative mortality and readmission rates could not be performed. Another limitation of our study is that a reliable long-term overall survival analysis could not be performed because the end date of the study was December 2023.

In conclusion, a high MFI-5 score is significantly associated with postoperative major complications in patients undergoing VATS-pulmonary resection with the diagnosis of NSCLC. Our results need to be supported by multicenter prospective studies.

## Data Availability

The data underlying this article will be shared on reasonable request to the corresponding author.
